# Primate-specific POTE-actin gene could play a role in human folliculogenesis by controlling the proliferation of granulosa cells

**DOI:** 10.1038/s41420-021-00566-1

**Published:** 2021-07-20

**Authors:** Yukiyo Kasahara, Satoko Osuka, Nobuyoshi Takasaki, Bayasula, Yoshihiro Koya, Natsuki Nakanishi, Tomohiko Murase, Tomoko Nakamura, Maki Goto, Akira Iwase, Hiroaki Kajiyama

**Affiliations:** 1 Department of Obstetrics and Gynecology, Nagoya University Graduate School of Medicine, 65 Tsurumai-cho, Showa-ku, Nagoya, 466-8550 Japan; 2 Department of Maternal and Perinatal Medicine, Nagoya University Hospital, 65 Tsurumai-cho, Showa-ku, Nagoya, 466-8550 Japan; 3 Bell Research Center for Reproductive Health and Cancer, Nagoya University Graduate School of Medicine, Nagoya, 466-8550 Japan; 4 Department of Obstetrics and Gynecology, Gunma University Graduate School of Medicine, 3-39-22 Showa-machi, Maebashi, 371-8511 Japan

**Keywords:** Cell growth, Reproductive disorders

## Abstract

Patients with primary ovarian insufficiency (POI) often have a high prevalence of autoimmune disorders. To identify antigenic molecules associated with ovarian autoimmunity, we performed immunoprecipitation (IP) screening using serum from patients with POI and the established human granulosa cell line (HGrC1). POTE ankyrin domain family member E (POTEE) and POTE ankyrin domain family member F (POTEF), proteins specific to primates, were identified as candidate antigens. Using immunohistochemistry (IHC) with human ovarian tissue, POTEE or POTEF was weakly seen in the granulosa cells (GCs) of primordial follicles and primary follicles, and strongly in large antral follicles and luteal cells. Interestingly, no signals were detected in growing GCs in secondary, preantral, and small antral follicles. Thus, to explore the function of POTEE and POTEF in human folliculogenesis, we established HGrC1 cell lines with drug-inducible expression of POTEF. Expression of POTEF significantly suppressed cell proliferation in HGrC1 cells. Furthermore, chaperonin containing TCP-1 complex (CCT) components, which affect folding proteins required for cell proliferation, was bound to the actin domain of POTEF protein. Although CCT is normally localized only around the Golgi apparatus, TCP-1α, a component of CCT, co-migrated closer to the cell membrane when POTEF expression was induced. These data suggest that the interaction between POTEF and CCT components impairs the usual function of CCT during cell growth. In addition, over-accumulation of POTEF in HGrC1 cells leads to autophagic failure. It was recently reported that knockout of an autophagic gene in mice leads to a phenotype similar to human POI. These results suggested that a proper amount of POTEF is required for the maintenance of GCs in follicle pools, whereas POTEF overaccumulation might be involved in follicle atresia and the development of POI. We also showed the possibility that POTEF could be an antigen involved in ovarian autoimmunity.

## Introduction

Ovarian reserve mainly depends on the number of remaining follicles in the ovary; early depletion of follicles results in primary ovarian insufficiency (POI). POI is defined as primary hypogonadism in a woman under the age of 40, mostly diagnosed by the presence of postmenopausal levels of follicle-stimulating hormone (>40 IU/L) in women under 40 years of age after 4 or more months of secondary amenorrhea [1–4]. POI affects ~1% of women before the age of 40 and 0.1% before the age of 30 [5].

Potential etiologies of POI that have been proposed include genetic abnormalities, metabolic or enzymatic dysfunction, infection, environmental factors, iatrogenic causes (radiotherapy or chemotherapy), and autoimmune causes [6, 7]. It is estimated that 10–40% of women with POI have another autoimmune disease, most commonly hypothyroidism (27%), followed by diabetes mellitus (2.5%) [8]. Patients with autoantibodies tend to have autoantibodies to multiple organs [9, 10]. Moreover, several lines of evidence currently point toward a link between autoimmunity and POI, including the presence of ovarian autoantibodies such as antibodies against the zona pellucida, oocyte cytoplasm, or entire ovary [11]. However, autoantibodies to granulosa cells (GCs) in patients with POI have not been well studied.

GCs have been shown to be important for follicle growth based on their responses to gonadotropins and crosstalk between oocytes [12, 13]. In this study, we first performed immunoprecipitation (IP) experiments with serum from patients with POI who have thyroid autoantibodies and with a human GC cell line (HGrC1) to screen for an autoantigen derived from GCs. Using proteomics analyses, we detected POTE ankyrin domain family member E (POTEE) or POTE ankyrin domain family member F (POTEF) in IP products as candidate autoantigens.

The POTE ankyrin domain gene family consists of 13 highly homologous variants dispersed among 8 different human chromosomes [14–17]. *POTE* genes are expressed in the testes, ovaries, placenta, and many cancers [14]. Interestingly, *POTEE* and *POTEF* genes on human chromosome 2 acquired the β-actin gene as a coding domain with retrotransposition only during primate evolution [18]. These two proteins that include the actin domain have 98.8% homology at the amino acid level. Recent studies have shown that the POTEE or POTEF protein has a pro-apoptotic function in vitro [19]. However, the in vivo functions of POTEE and POTEF remain unknown. We can observe the signals of POTEE or POTEF in normal human ovaries with immunohistochemistry (IHC). To identify the roles of POTEE or POTEF in human folliculogenesis, we established HGrC1 lines that express POTEF with chemical induction. POTEF can repress cell proliferation in vitro through the binding of chaperonin containing TCP-1 (CCT) complex, suggesting that POTEF could arrest the growth of GCs in early folliculogenesis for the preservation of primordial follicle endowment. Moreover, overaccumulation of POTEF in cells with impairments in the autophagy system could contribute to the onset of POI or cell death in atretic follicles and the corpus luteum in a dose-dependent manner.

## Results

### Screening for autoantibodies in the serum of patients with POI who have thyroid autoantibodies

We first tried determining whether anti-ovarian autoantibodies are found in the serum of patients with POI who have thyroid autoantibodies (POI Ab+). Using IHC for human ovarian sections, serum from some patients with POI and thyroid autoantibodies showed immunoreactivity to GCs in human ovaries, whereas there was no immunoreactivity to serum from women who had regular menstrual cycles (Supplementary Fig. 1A).

To identify candidate ovarian autoantigens reactive to autoantibodies in the serum of patients with POI who have thyroid autoantibodies, we designed IP experiments using serum from patients with POI with thyroid autoantibodies, without thyroid autoantibodies, and control patients using proteins from the human GCs line HGrC1. We subsequently analyzed the precipitated proteins using mass spectrometry (Supplementary Fig. 1B). Fifty-two proteins were detected in samples from patients with POI who have thyroid autoantibodies. Among these molecules, 20 were detected in 2 or 3 POI Ab+ patient samples (Supplementary Fig. 1C, Supplementary Table 1). Mass spectrometry revealed that the 120-kDa precipitated autoantigen was POTEE or POTEF.

### POTEE or POTEF expression depends on the developmental stage of the ovarian follicle

**Fig. 1:**
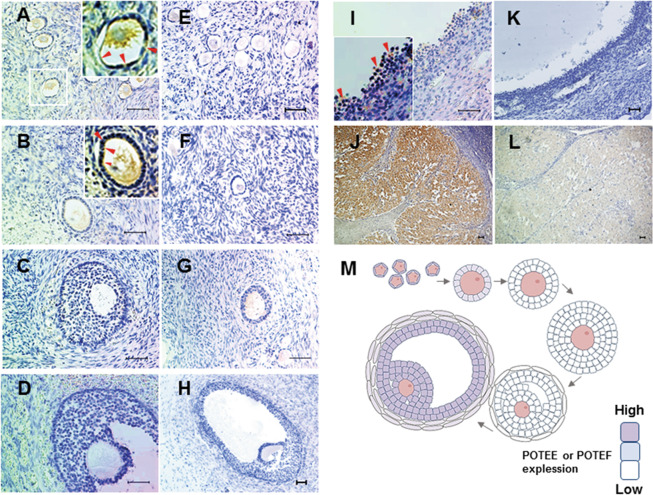
Localization of POTEE or POTEF protein during human folliculogenesis. Representative IHC images of POTEE or POTEF staining in serially sectioned human normal ovarian tissue. **A** Primordial follicle. **B** Primary follicle. **C** Secondary follicle. **D** Small antral follicle. **I** Large antral follicle. **J** Corpus luteum. **E**–**H**, **K**, **L** IHC images with rabbit IgG was used as a negative control. Scale bars, 50 µm. **M** Illustration of changes in POTEE or POTEF protein expression during follicle development. Red arrowheads indicate POTEE or POTEF staining in granulosa cells.

To ascertain whether POTEF or POTEE is actually expressed in the human ovary, we performed IHC with normal human ovarian tissue. Unfortunately, the commercial antibody recognized both recombinant POTEE and POTEF protein with western blotting (Supplementary Fig. 2A). Therefore, the signal of the antibody used in IHC experiments was not able to distinguish between POTEE and POTEF. Signals for POTEE or POTEF antibodies were observed in stroma cells and the cytoplasm of the oocytes (Fig. 1 A–D). These signals could represent background signals because they were frequently found in stroma cells and the cytoplasm of the oocytes with rabbit IgG in a negative control (Fig. 1 E–H). Meanwhile, specific weak signals for POTEE or POTEF were detected in the GCs of primordial follicles and primary follicles (Fig. 1A, B). The signals seemed to localize close to the plasma membrane in the GCs of primordial and primary follicles, as previously reported [19]. In contrast, they were hardly observed in growing secondary, preantral, and small antral follicles (Fig. 1C, D). Interestingly, strong signals were also found in the GCs of large antral follicles, which are considered preovulatory follicles, and in luteal cells after ovulation (Fig. 1I, J). Several GCs in primordial follicles are required for the formation of one follicle, but the number increases to 10^6^ to 10^7^ cells in the preovulatory follicle stage [20]. Because there was no signal against POTEE or POTEF in the GCs of secondary follicles and small antral follicles (Fig. 1C, D), we presumed that POTEE or POTEF might contribute to the growth of GCs under a gonadotropin-independent manner. In addition, after the completion of the follicle growth before ovulation and in the corpus luteum after ovulation, POTEE or POTEF is expected to function again in human GCs.

### Establishment of HGrC1 cell lines that express POTEF with chemical induction

**Fig. 2:**
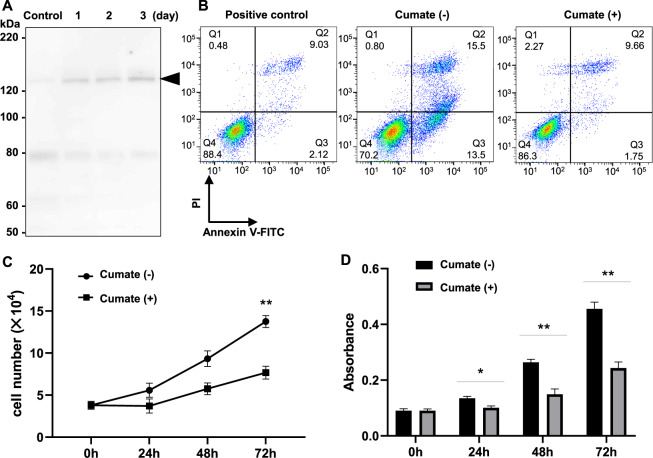
Effects of drug-induced expression of POTEF protein in HGrC1 cells. **A** Western blotting with anti-Flag antibody. In the HGrC1 cell line with a chemical inducible expression vector for POTEF tagged with Flag (CuO-POTEF-Flag HGrC1), POTEF protein (arrowhead) was detected for 3 days when the cumate chemical solution was present. **B** Apoptosis of CuO-POTEF-Flag HGrC1 cells treated with or without cumate treatment for 72 h, stained with fluorescein isothiocyanate (FITC)-conjugated annexin V, and analyzed using flow cytometry. Positive control refers to the rate of apoptosis in HGrC1 cells cultured without serum for 72 h. **C** Analysis of the number of CuO-POTEF-Flag HGrC1 cells counted every 24 h, with or without cumate treatment. **D** Bromodeoxyuridine (BrdU) proliferation assay of CuO-POTEF-Flag HGrC1 cells with or without cumate treatment. The incorporation of BrdU was measured using an ELISA system. Data are presented as means ± SD. Three independent experiments showed similar results. *P < 0.05 and **P < 0.01 with vs without cumate treatment.

Reverse transcription-polymerase chain reaction (RT-PCR) detected POTEF transcripts in the HGrC1 cell line and in primary human GCs, considered to be early luteinized GCs (Supplementary Fig. 2B). Thus, the expression of *POTEF* gene is activated in human GCs and HGrC1 cells. However, POTEE or POTEF protein was not detected in HGrC1 cells at the western blot level using a commercial antibody. Because the HGrC1 cell line was established from GCs in secondary follicles [21], this result is consistent with the absence of POTEE or POTEF protein signals in the GCs of secondary follicles, as shown in Fig. 1. To confirm the hypothesis that POTEE or POTEF controls apoptosis or proliferation of GCs in folliculogenesis, we tried to establish HGrC1 cells in which POTEE or POTEF protein expression occurs upon chemical induction. In this study, we focused on POTEF. We established two independent HGrC1 cell lines that expressed drug-inducible POTEF protein tagged with DDDDK (CuO-POTEF-Flag HGrC1). Without chemical induction, the expression of POTEF protein in CuO-POTEF-Flag HGrC1 cells was hardly observed with western blotting using an anti-DDDDK antibody (control lane in Fig. 2A). In contrast, CuO-POTEF-Flag HGrC1 cells in culture continuously expressed POTEF protein for 1–3 days when the chemical was added (Fig. 2A).

### POTEF expression suppresses HGrC1 cell proliferation and does not cause apoptosis in HGrC1 cells

It was previously reported that POTEF expression induces apoptosis of cultured cells [19]. Thus, we investigated whether POTEF expression induces apoptosis in HGrC1 cells. Annexin V expression was measured by flow cytometry. There were no remarkable changes in the rate of apoptosis in CuO-POTEF-Flag HGrC1 cells during POTEF expression (cumate (+) column, Fig. 2B). These results indicated that POTEF does not produce programmed cell death, at least in a HGrC1 cell line derived from human GCs.

Next, we examined the possibility that POTEF affects cell proliferation. Although the number of HGrC1 cells usually increases about threefold after 72 h of culture in the absence of chemical induction, the number of cells after 72 h of culture was suppressed ~1.5-fold with continuous POTEF expression (cumate (+), Fig. 2C). The MTS assay showed that continuous POTEF expression suppresses cell growth (Fig. 2D). These results suggested that POTEF could attenuate the proliferation of HGrC1 cells, but not induce apoptosis. These findings support the possibility that POTEF arrest the growth of GCs within primary follicles. It was also observed that POTEF expression inhibits the growth of HEK293T cells, similar to HGrC1 (Supplementary Fig. 3).

### Identification of molecules interacting with POTEF in HGrC1 cells

**Fig. 3:**
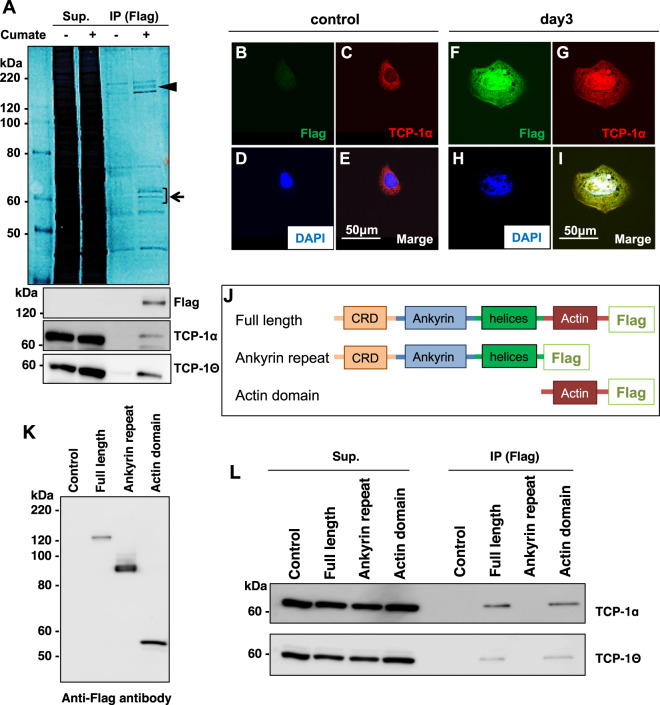
POTEF interacts with CCT subunits through its own actin domain. **A** Co-immunoprecipitants with anti-Flag antibody were separated on SDS-PAGE and visualized using Negative Gel Stain MS Kit. The band around 120 kDa (arrowhead) was subsequently confirmed as POTEF with western blotting. Other unique bands visualized around 60 kDa (arrow) were identified with mass spectrometry (Supplementary Table 2). Two CCT subunits were confirmed as molecules interacting with POTEF using both anti-TCP-1α and anti-TCP-1θ antibodies. **B**–**D** Fluorescent image of CuO-POTEF-Flag HGrC1 cells with anti-Flag antibodies, anti-TCP-1α antibodies, or DAPI nuclear staining, respectively, without cumate treatment. **E** Merged image of (**B**), (**C**), and (**D**). **F**–**H** Fluorescent image of CuO-POTEF-Flag HGrC1 cells with anti-Flag antibodies, anti-TCP-1α antibodies, or DAPI nuclear staining, respectively, after 3 days in the presence of cumate solution. **I** Merged image of (**F**), (**G**), and (**H**). Scale bars, 50 µm. **J** Schematic comparison of human POTEF based on the immunoprecipitation experiments. Full-length POTEF has a cysteine-rich domain (CRD, orange), ankyrin repeat (Ankyrin, blue), helices domain (helices, green), actin domain (Actin, red), and Flag tag at the C-terminus. The ankyrin repeat consisted of full-length POTEF without the actin domain. The actin domain consisted of only the actin domain. **K** Immunoprecipitation with anti-Flag antibody followed by western blotting with anti-Flag antibody. **L** Co-Immunoprecipitation with anti-Flag antibody followed by western blotting with anti-TCP-1α and anti-TCP-1θ antibodies.

POTEF and POTEE each contain four domains, including an actin domain (Fig. 3J) [18]. However, the functions of each domain are unknown. To date, molecules that directly interact with POTEF protein have not been reported. Hence, to assess the molecular role of POTEF in the suppression of cell proliferation, we performed IP using an anti-DDDDK antibody to identify molecules interacting with POTEF. SDS-polyacrylamide gel electrophoresis (SDS-PAGE) staining identified several bands around 120 and 60 kDa, respectively (Fig. 3A). The 120-kDa molecule (arrowhead in Fig. 3A) was subsequently confirmed to be POTEF with western blotting. Regarding the bands around 60 kDa (arrow in Fig. 3A), mass spectrometry detected the subunits of CCT (Supplementary Table 2) The IP-western blot with antibodies against each subunit of CCT, anti-TCP-1α and anti–TCP-1θ, indicated that POTEF interacts with CCT subunits (Fig. 3A).

To confirm whether CCT interacts with POTEF in HGrC1 cells, we performed fluorescent staining of CuO-POTEF-Flag HGrC1 cells using an anti-TCP-1α antibody. When POTEF was not expressed, TCP-1α was localized to the cytoplasm around the nucleus (Fig. 3B–E). This result was consistent with the previous report that CCT is associated with the cytoplasmic aspect of membranes of the trans-Golgi network [22]. With chemical induction, POTEF co-localized with TCP-1α around the nucleus. However, POTEF was also localized close to the plasma membrane, as previously reported [19]. Interestingly, TCP-1α became localized cytoplasm and close to the plasma membrane with co-localization with POTEF (Fig. 3F–I). This result supports that CCT, or at least TCP-1α, directly binds to POTEF protein and migrates close to the plasma membrane as these two molecules interact.

### CCT binds to the actin domain of POTEF protein

It has been reported that CCT contributes to the folding of cytoskeletal proteins including tubulin and actin as a cytosolic chaperone [23, 24], thereby regulating cell proliferation [25]. To ascertain whether CCT might bind to the actin domain of POTEF protein, we produced three types of proteins: full-length POTEF-Flag (full length), partial POTEF without the actin domain-Flag (Ankyrin repeat), and only the actin domain-Flag (actin domain) in HEK293T cells (Fig. 3J, K). We performed IP-western blot experiments with each constructed protein. Western blotting with two antibodies showed that TCP-1α and TCP-1θ bind the actin domain of POTEF (Fig. 3L). These results suggest that POTEF would block the role of CCT in cell proliferation through associations between the actin domain of POTEF and CCT.

### Impairment of CCT attenuates the proliferation of HGrC1 cells

**Fig. 4:**
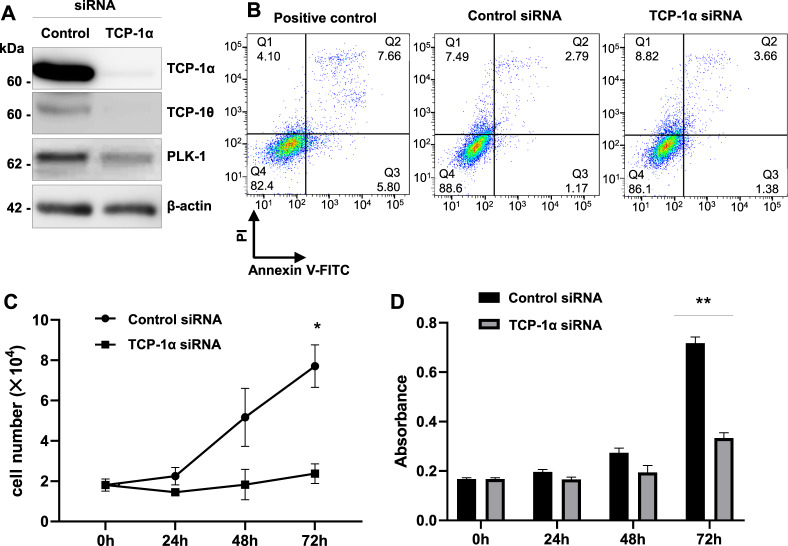
Effects of inhibiting CCT function with siRNA against TCP-1α in HGrC1 cells. **A** Relative protein expression levels were determined with a western blot assay. Transfection of TCP-1α siRNA into HGrC1 cells reduced the expression of TCP-1α; TCP-1θ, another subunit of CCT; and PLK1, a cell growth factor. β-Actin was used as the control. **B** Alteration in apoptosis of HGrC1 cells with TCP-1α siRNA or control siRNA. Cells were stained with fluorescein isothiocyanate (FITC)-conjugated annexin V and analyzed using flow cytometry. Positive control refers to the rate of apoptosis in HGrC1 cells cultured without serum for 72 h. **C** Analysis of the number of CuO-POTEF-Flag HGrC1 cells treated with TCP-1α siRNA or control siRNA. Cells were counted every 24 h. **D** BrdU proliferation assay of CuO-POTEF-Flag HGrC1 cells with TCP-1α siRNA or control siRNA. BrdU incorporation was measured using an ELISA system. Data are presented as means ± SD. Three independent experiments showed similar results. *P < 0.05 TCP-1α siRNA vs control siRNA.

It has been demonstrated that depletion of CCT strongly arrests cell growth [26]. To confirm the contribution of CCT to cell proliferation, we employed the siRNA approach against the TCP-1α subunit of CCT in HGrC1 cells. siRNA expectedly down-regulated not only levels of TCP-1α protein, but also of TCP-1θ, another subunit of CCT (Fig. 4A), suggesting that at least one subunit of CCT, i.e., TCP-1α, is essential for the maintenance of CCT formation. In addition, levels of polo-like kinase 1 (PLK1) protein, which has been reported to be necessary for the biogenesis and mitotic progression as a substrate of CCT, were slightly reduced [25]. These results suggested that siRNA specific to TCP-1α leads to CCT dysfunction.

We evaluated whether knockdown of TCP-1α gives rise to apoptosis or decreased cell proliferation of HGrC1 cells. Knockdown of TCP-1α had no effect on the apoptosis rate in HGrC1 cells (Fig. 4B). However, cell counts experiments and the MTS assay clearly indicated that knockdown of TCP-1α suppresses the proliferation of HGrC1 cells (Fig. 4C, D). These results support the idea that POTEF inhibits cell growth by interfering with the role of CCT in cell proliferation through direct interaction between the two molecules.

### POTEF does not affect CCT expression

**Fig. 5:**
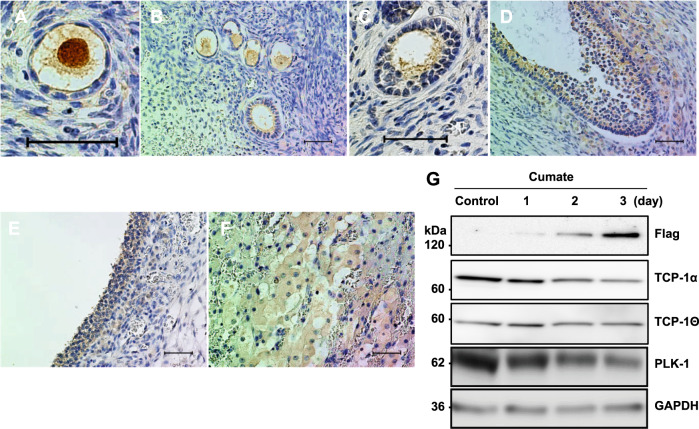
Expression of TCP-1α in human granulosa cells in vivo and in vitro. Representative IHC images of TCP-1α staining in serially sectioned human normal ovarian tissue. **A** Primordial follicle. **B** Primary follicle. **C** Secondary follicle. **D** Small antral follicle. **E** Large antral follicle. **F** Corpus luteum. Scale bars, 50 µm. **G** Relative levels of target proteins in CuO-POTEF-Flag HGrC1 cells with POTEF expression. Cell lysates were western blotted with anti-Flag, anti-TCP-1α, anti-TCP-1θ, and anti-PLK1 antibodies. Amounts of two CCT component proteins and PLK1 did not drastically change with the presence of POTEF protein. GAPDH was used as the control.

Although the expression pattern of CCT has already been evaluated in rodent spermiogenesis [27], there are no descriptions of CCT expression levels in ovarian GCs or the role of CCT in human folliculogenesis. To evaluate TCP-1α expression in human ovarian tissues, we perform IHC using an anti-TCP-1α antibody. TCP-1α localized to the cytoplasm of GCs during each stage of folliculogenesis (Fig. 5A–F), suggesting that POTEF in the GCs of primordial and primary follicles would have no effect on the expression of TCP-1α during human folliculogenesis. Actually, induced expression of POTEF had almost no effect on levels of TCP-1α, TCP-1θ, and PLK1 in HGrC1 cells (Fig. 5G). However, signals of TCP-1α localized close to the plasma membrane in the GCs of primordial and primary follicles, along with signals of POTEE or POTEF (Fig. 1A, B and Fig. 5A, B). Meanwhile, TCP-1α was distributed in the cytoplasm of growing secondary, preantral, and small antral follicles. No signals were detected with an anti-POTEE or POTEF antibody (Fig. 1C, D and Fig. 5C, D). These observations assume that POTEF inhibits cell proliferation in primordial and primary follicles by shifting the cellular localization of CCT through direct interactions, without changing CCT expression levels.

### Overaccumulation of POTEF results in autophagic failure in HGrC1 cells

Because the strength of signals with anti-POTEE or POTEF antibodies seemed to be higher in the GCs of large antral follicles and in luteal cells after ovulation (Fig. 1I, J), we suspected that POTEF might have a dose-dependent effect on the growth of GCs during the different stages of folliculogenesis. Thus, we tried to observe alterations in CuO-POTEF-Flag HGrC1 cells with overaccumulation of POTEF over 6 days of incubation with continuous chemical treatment.

**Fig. 6:**
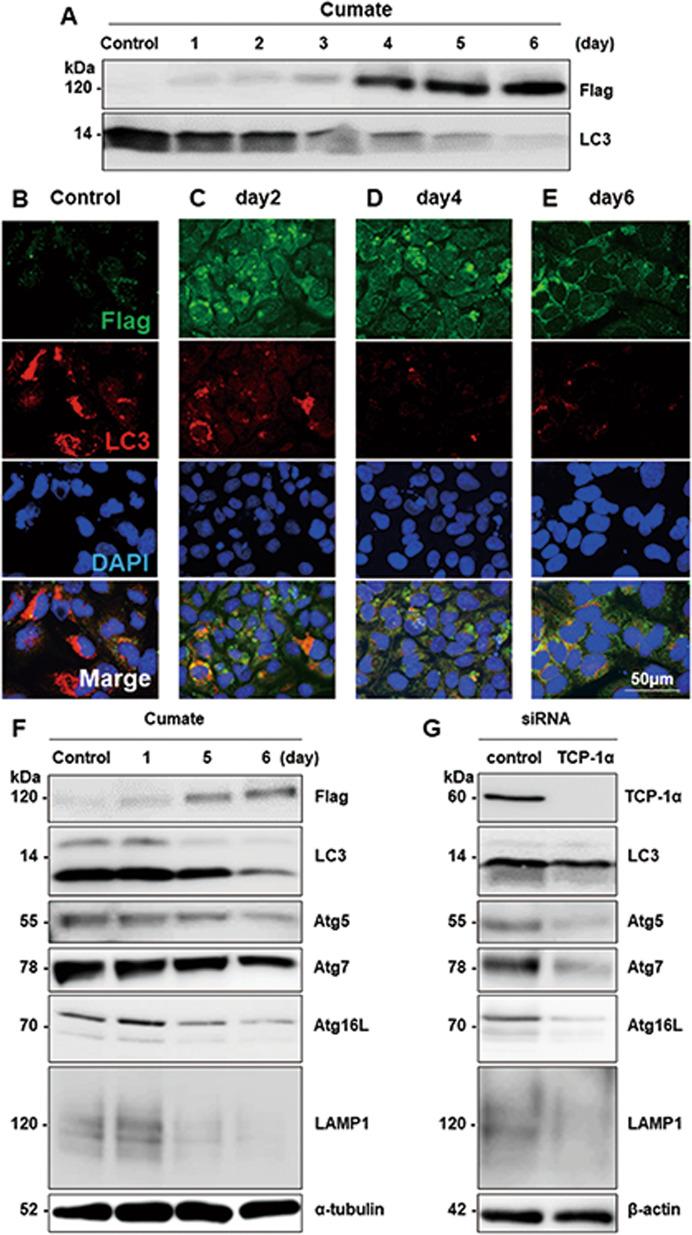
Influence of persistent POTEF protein expression in HGrC1 cells. **A** Continuous culture of CuO-POTEF-Flag HGrC1 cells with cumate solution for 6 days. POTEF expression was detected persistently for 6 days and excessively after 4 days. LC3 expression decreased with persistent POTEF expression in CuO-POTEF-Flag HGrC1 cells. **B**–**E** Fluorescent staining of CuO-POTEF-Flag HGrC1 cells with anti-LC3 antibody with continuous POTEF expression. **F** With over-accumulation of POTEF in CuO-POTEF-Flag HGrC1 cells, levels of autophagy-related factors Atg5, Atg7, and Atg16L were slightly decreased. LAMP1, a lysosomal marker, was hardly detected with western blotting after 6 days. α-tubulin was used as the control. **G** Knockdown of TCP-1α using siRNA in HGrC1 cells slightly decreased autophagy-related factors, except for LC3. Reduction in LAMP1 expression was remarkable. β-Actin was used as the control.

It was recently reported that germ cell-specific knockout of the essential autophagy induction gene Atg7 in mice resulted in severe ovarian follicle loss that is very similar to human POI [28]. Accordingly, we focused on molecules contributing to the autophagy system with POTEF over-accumulation in HGrC1 cells. Continuous expression of POTEF apparently decreased LC3 a marker of autophagy reflecting intracellular autophagic activity (Fig. 6A). Fluorescent staining of CuO-POTEF-Flag HGrC1 cells showed less LC3 immunoreactivity with POTEF over-accumulation (Fig. 6B–E). Accumulation of POTEF slightly decreased levels of autophagy-related factors such as Atg5, Atg7, Atg16L and the lysosomal marker LAMP1 (Fig. 6F). These results suggest that POTEF overexpression could damage the autophagy system. Interestingly, except for LC3, knockdown of TCP-1α by siRNA modestly decreased Atg5, Atg7, Atg16L, and LAMP1 levels in HGrC1 cells overexpressing POTEF (Fig. 6F, G). These results are consistent with a previous report showing that autophagic flux is reduced by compromise of individual CCT subunits [29]. These results indicate that POTEF over-accumulation with CCT impairment in the human ovary could contribute to the development of POI through failure of the autophagy system. It is likely that overaccumulation of POTEF might be involved in the degeneration of the corpus luteum through autophagy system dysfunction.

## Discussion

In this study, we identified POTEF and POTEE as candidate proteins contributing to ovarian autoimmunity based on proteomic analysis of IP products in the serum of patients with POI. Furthermore, we firstly succeeded in the detection of POTEE or POTEF expressions in human folliculogenesis. Using in vitro experiments with a human GC line, we found that POTEF repressed the proliferation of HGrC1 through interactions with the components of CCT. We also observed that over-accumulation of POTEF induces autophagy incompetence in HGrC1 cell line.

To date, few previous studies have reported the in vivo functions of the POTE gene family. Of note, the *POTEE* and *POTEF* genes coding a β-actin gene emerged during primate evolution [18], but it was difficult for us to define the functions of POTEE or POTEF in humans. In this study, we detected POTEE or POTEF in human ovaries using IHC. Our results indicated that the expression levels of POTEE or POTEF vary according to follicle development stage. Notably, POTEE or POTEF were not expressed in follicle stages with dramatic growth that requires high proliferative activity in GCs. These findings suggest that POTEE or POTEF could contribute to the growth of GCs in early follicles, and that these might be involved in the regulation of single ovulation specifically in primates.

Because there is 98.8% homology between POTEE and POTEF at the amino acid level, POTEE and POTEF are considered to have similar functions. Our results indicated that CCT recognizes and binds to the actin domain of POTEF protein. As a result of the actin domain, *POTEE* and *POTEF* genes would possess functions different from the functions of other genes in the POTE family that do not encode an actin domain. In the near future, it is necessary to confirm whether genes in the POTE family not encoding an actin domain can regulate cell proliferation using the chemical induction system we used to validate gene function.

We established a drug-induced expression system that enabled sustained expression of POTEF in HGrC1 cells. Contrary to previous reports with HeLa cells [19], we could not detect markers of apoptosis in HGrC1 cells using our induction system. Comparing the amount of POTEF after 4 days induction, it was uniformly modest in culture for 3 days (Fig. 6). This appropriate amount would be effective in inhibiting the growth of GCs in primordial and primary follicles by blocking the function of CCT.

Moreover, the results of immunofluorescence staining indicated that induced POTEF expression is colocalized with TCP-1α in the vicinity of the cell membrane in HGrC1 cells. According to a previous report, CCT is associated with the cytoplasmic aspect of membranes in the trans-Golgi network [22]. Thus, it was interesting that the localization of TCP-1α with POTEF changed from close to the nucleus to near the plasma membrane (Fig. 3G). It is likely that the accumulation of these molecules around the plasma membrane prevented the migration or localization of receptors for follicle-stimulating hormones or receptors for cell growth signals in the GCs of early follicles. It would be useful to confirm the localization of signal receptors when POTEF is expressed in HGrC1 cells.

The knockdown of TCP-1α with specific siRNA had no effects on signals of LC3, a marker of autophagic activity (Fig. 6G). Our data were consistent with findings from a previous report [29] that showed CCT impairment blocks the degradation of LC3. Over-accumulation of POTEF in HGrC1 cells resulted in the decline of LC3 signals. Taken together, there might be another mechanism that blocks the autophagy system other than over-accumulation of POTEF and knockdown of CCT. To define the in vivo functions of CCT in folliculogenesis, using the knockdown technology in rodents might be effective.

We speculated that over-accumulation of POTEF in the GCs of primordial and primary follicles contributes to the onset of POI through the dysfunction of the autophagic machinery. Germ cell-specific mutation of the *Atg7* gene in mice leads to a human POI phenotype [28]. Sequence analysis of exomes in women with POI revealed the functional link between variants of autophagy-related genes (ATGs) and POI, and that these variant ATGs led to impairment of autophagy [30]. Understanding the molecular mechanisms by which over-accumulation of POTEF leads to damage of the autophagy system can shed light on the development of POI.

It remains unknown whether the accumulation of POTEE or POTEF affects the GCs of large antral follicles before ovulation or luteal cells after ovulation. The relationship between autophagy or apoptosis and human ovarian physiology has been described. Kang et al. found that the absence of LC3 in human cumulus cells leads to cell death [31]. Another autophagic molecule could participate in the cellular lifespan of human corpus luteum cells through the retention of autophagy [32]. POTEE and POTEF could contribute to the autophagy system and maintenance of a functional human ovary. At present, it is unclear how POTEE or POTEF molecules are recognized as autoantigens. To better understand human ovarian function, it might be useful to titrate antibodies against POTEE or POTEF as an immune autoantigen in the serum of women, including patients with POI.

## Materials and methods

### Human subjects

This study was approved by the ethics committee of the Nagoya University Graduate School of Medicine (Reference number: 2013-0265). In accordance with the Declaration of Helsinki, all patients provided written informed consent prior to participation.

Blood samples were obtained from the patients with POI and normal controls with regular menstrual cycles. Serum was separated from whole blood and stored at −80 °C until analysis. The background characteristics of patients whose serum samples were used in the screening for ovarian autoantibodies are described in Supplementary Table 1.

For IHC experiments, samples of surgically removed ovarian tissue were obtained from six women of reproductive age (19–36 years). Patients were selected based on a history of regular menstrual cycles and no intrauterine device use or hormonal therapy for more than 6 months prior to surgery. The six women who participated in the study were patients undergoing surgical procedures for cervical cancer.

### Immunohistochemistry

Ovarian tissue samples were fixed in 10% neutral buffered formalin and embedded in paraffin. Immunohistochemical staining was performed using the avidin-biotin immunoperoxidase method and the Histofine SAB-PO kit (Nichirei Biosciences Inc., Tokyo, Japan) according to the manufacturer’s protocol. Endogenous peroxidase activity was blocked by 3% hydrogen peroxide in methanol for 15 min. For screening of ovarian autoantibodies in the serum of patients with POI, blocking was performed with 10% normal goat serum in phosphate-buffered saline (PBS). Tissue sections were incubated at 4 °C overnight with serum of patients with POI with thyroid autoantibodies or women with normal menstrual cycles. Goat anti-Human IgG + IgM + IgA conjugated with biotin (ab102418, Abcam, Cambridge, UK) was used as the secondary antibody. For staining of POTEF and TCP-1α, nonspecific IgG binding was blocked using 10% normal rabbit serum in PBS. Tissue sections were incubated at 4 °C overnight with an anti-POTEE antibody (ab108190, 1:100 dilution, Abcam) and anti-TCP-1α antibody (sc-374088, 1:100 dilution, Santa Cruz Biochemistry, Dallas, TX, USA). Rabbit immunoglobulin G (IgG) was used as a negative control. Slides were counterstained with Meyer’s hematoxylin (Wako Pure Chemical Industries, Ltd, Osaka, Japan) at room temperature for 1 min. The slides were viewed using BZ9000 microscope (Carl Zeiss Microscopy Co., Ltd., Oberkochen, Germany).

### Cell culture

According to our previous paper [21], human nonluteinized granulosa cells (HGrC1 cells) were cultured at 37 °C in Dulbecco’s modified Eagle’s medium (DMEM, Sigma-Aldrich, St. Louis, MO, USA) supplemented with 10% fetal bovine serum (FBS, Sigma), 100 IU/ml of penicillin, 100 µg/ml of streptomycin, and 25 mg/l of amphotericin B at 5% CO_2_ and 5% O_2_. 293T cells were also maintained in DMEM containing 10% FBS and 100 IU/ml of penicillin-streptomycin at 37 °C and 5% CO_2_. Before starting the experiment, it was confirmed that there was no contamination with mycoplasma.

### RT-PCR

Total RNA was isolated from HGrC1 cells or primary cultured luteinized GCs obtained from in vitro fertilization in 35-mm dishes using the RNeasy Mini kit (QIAGEN, Inc., Valencia, CA, USA) following the manufacturer’s protocol. A reverse transcriptase reaction with 1 μg of total RNA was carried out with a first-strand cDNA synthesis kit (ReverTra Ace α; TOYOBO Co., Ltd., Osaka, Japan). Thereafter, 1-μl aliquots of the RT reaction products were used for PCR to amplify mRNA from the entire POTE family or only POTEF mRNA. The following sets of oligonucleotide primers used: T444, 5′-CAATGCCAGGAAGATGAATGTGCG-3′; T445, 5′-TCTCTGGCCGTCTGTCCAGATAGA T-3′; POTEFF, 5′-TTGTCCACCGCAAATGCTTG-3′; and POTEFR, 5′-AAGCTTCCTCCAACCGACTG-3′. Amplification was performed using Taq polymerase (PerkinElmer, Waltham, MA, USA) over 35 cycles. Each cycle consisted of denaturation at 94 °C for 1 min, annealing at 54 °C for 1 min, and extension at 72 °C for 1 min. We compared the expression levels determined by RT-PCR with expression levels in SKOV-3 cells derived from ovarian adenocarcinoma as a positive control and distilled water as a negative control.

### Establishment of the CuO-POTE-Flag HGrC1 and HEK293T cell lines, which express POTEF tagged with DDDDK upon chemical induction

The DDDDK-tagged human POTEF cDNA clone RC216378 was purchased from OriGene Technologies, Inc. (Rockville, MD, USA). For the establishment of HGrC1 and HEK 293T cell lines expressing POTEF, DDDDK-tagged human POTEF cDNA was subcloned into Enhanced Episomal Vector cloning and expression vector formats, which feature an ultra-tight cumate inducible system (catalog#EEV61A-1, System Biosciences, Palo Alto, CA, USA). Avalanche-Omni Transfection Reagent (EZ Biosystems, College Park, Maryland, USA) was employed as the DNA transfection reagent for the HGrC1 cell line. HGrC1 cells with the transfected DNA were screened and maintained with 8 mg/ml of puromycin (InvivoGen, Carlsbad, CA, USA).

### Western blot analysis

CuO-POTE-Flag HGrC1 cells were cultured in DMEM with 10% FBS for 24 h. The cells were then treated in DMEM with 0.1% cumate solution (QM150A-1, System biosciences) for 1–6 days. After treatment, cells lysates were resolved in SDS-PAGE and transferred onto an Immune-Blot PVDFmembrane (BIO-RAD, Richmond, CA, USA) for immunoblotting. Membranes were immunoblotted with an anti-DYKDDDDK tag antibody (#014-22383, Fujifilm Wako Pure Chemicals, Osaka, Japan, 1:2000), anti-TCP-1α antibody (sc-374088, Santa Cruz Biotechnology, Inc. 1:200), anti-TCP-1θ antibody (sc-377261, Santa Cruz Biotechnology, Inc. 1:200), anti-PLK1 antibody (sc-53751, Santa Cruz Biotechnology 1:500), anti-LC3 antibody (M186, MBL, Nagoya, Japan, 1:5000), anti-Atg5 antibody (#12994, Cell Signaling, Danvers, MA, USA, 1:1000), anti-Atg7 antibody (#8558, Cell Signaling, 1:1000), anti-Atg16L (M150-3MS, MBL,1:1000), anti-LAMP1 antibody (sc-20011, Santa Cruz Biotechnology, Inc. 1:200), anti-GAPDH antibody (M171-7, MBL, 1:5000), anti–β-actin antibody (#017-24573, Fujifilm Wako Pure Chemicals, 1:3000) and anti–α-tubulin antibody (PM054, MBL 1:5000).

### Knockdown of TCP-1α using siRNA

For siRNA experiments, HGrC1 cells were transfected with 5 μM of TCP-1α siRNA (cat#;4427037, Life Technologies/Ambion, Waltham, MA, USA) and 5 μM of Stealth RNAi siRNA Negative Control as a negative control (no. 12935114, Life Technologies/Invitrogen) for 72 h using Lipofectamine RNAi Max (Life Technologies/Invitrogen) following the manufacturer’s instructions. The culture medium and whole-cell extracts were obtained for biochemical analyses.

### Apoptosis assay

To examine changes in apoptosis rates associated with POTEF expression in HGrC1 cells, CuO-POTEF-Flag HGrC1 cells were treated with cumate solution for 3 days. As a negative control, CuO-POTEF-Flag HGrC1 cells cultured without cumate solution were used. Cells obtained by culturing CuO-POTEF-Flag HGrC1 in a serum-free culture medium for 2 days were used as a positive control. Apoptosis was measured based on fluorescein isothiocyanate (FITC)-conjugated annexin V staining using a MEBCYTO Apoptosis Kit (MBL) according to the manufacturer’s recommendations and flow cytometry.

### Cell count experiments

CuO-POTEF-Flag-HGrC1 cells were plated at 30 × 10^3^ in each well of 24-well plates with or without cumate solution for 72 h. For siRNA experiments, HGrC1 cells were plated at 30 × 10^3^ in each well of 24-well plates and cultured with 5 μM of TCP-1α siRNA or 5 μM of Stealth RNAi siRNA Negative Control for 72 h. In both cases, the number of cells was counted every 24 h.

### MTS assay

Cells were seeded into a 96-well plate at a density of 3,000 cells per well for 72 h. Cell viability was measured after 0, 24, 48, and 72 h using 3-(4,5-dimethylthiazol-2-yl)-5-(3-carboxymethoxyphenyl)-2-(4-sulfophenyl)-2H-tetrazolium inner salt (MTS) according to the manufacturer’s instructions (Promega Corporation, Madison, WI, USA). Absorbance was measured at a wavelength of 490 nm with a Viento 808 IU absorbance reader (BioTek, Winooski, VT, USA).

### Statistical analysis

All experimental data are presented as means ± SEM. *F*-tests were used to check the equality of two variances. Student’s *t*-test was used to compare means between two groups. All statistical analyses were performed using GraphPad Prism8 software (GraphPad Software Inc., San Diego, CA, USA). *P* < 0.05 was considered statistically significant.

### Immunoprecipitation

To screen for autoantigens of ovarian autoantibodies, IP was performed using patient samples. This included patients with POI who had thyroid antibodies, patients with POI who did not have thyroid autoantibodies, and control patients (Supplementary Table 1). Serum and cell lysates of HGrC1 cells with Dynabeads Protein G (Thermo Fisher Scientific, Waltham, MA, USA) were also used. The antigen-antibody complex was analyzed by mass spectrometry.

To identify molecules interacting with POTEF, CuO-POTEF-Flag HGrC1 cells were cultured for 2 days in DMEM medium with cumate solution. The cell lysates were used for IP.

IP was carried out using the DDDDK-tagged Protein Magnetic Purification Kit (MBL). The purification products were loaded in an SDS-PAGE gel and protein bands on the SDS-PAGE gel were visualized using the Negative Gel Stain MS Kit (Fujifilm Wako Pure Chemical Corporation). Unique bands visualized around 60 kDa on the SDS-PAGE gel were cut out, shredded, bleached, and digested with trypsin. The peptide sequences were analyzed with mass spectrometry.

### Mass spectrometry

The proteins of the antigen-antibody complex or bands on SDS-PAGE gel were digested with trypsin for 16 h at 37 °C after reduction and alkylation. To screen for autoantigens of ovarian autoantibodies, the proteins of the antigen-antibody complex were eluted using an alkylation solution by adding 10 μl of 0.1 M dithiothreitol and spraying N_2_ for 1 min. The samples were left at room temperature for 30 min and then alkylated in 10 μl of 0.2 M iodoacetamide for 1 h. The proteins were collected using chloroform-methanol precipitation at room temperature in the dark. The dried samples were added to 10 μl of 6 M urea and 40 μl of 0.1 M Tris-Cl, digested with a trypsin solution, and incubated for 16 h at 37 °C.

Nanoelectrospray tandem mass analysis was performed using an LTQ Orbitrap XL mass spectrometry system (Thermo Fisher Scientific Inc.) combined with a Paradigm MS4 HPLC System (Michrom BioResources Inc., Auburn, CA, USA). Samples were injected into the Paradigm MS4 HPLC System equipped with an L-column2 ODS that was 0.1 mm in diameter and 150 mm in length (Chemicals Evaluation and Research Institute, Tokyo, Japan). Reversed-phase chromatography was performed with a linear gradient (0 min, 5% B; 100 min, 50% B) of solvent A (2% acetonitrile with 0.1% formic acid) and solvent B (90% acetonitrile with 0.1% formic acid) at an estimated flow rate of 500 nl/min. Ionization was performed with an ADVANCE Spray Source (Michrom BioResources Inc.) with a capillary voltage at 1.7 kV and temperature of 150 °C. A precursor ion scan was carried out using a 400–2000 mass to charge ratio (*m/z*) prior to MS/MS analysis. Multiple MS/MS spectra were submitted to the Mascot program, version 2.5.1 (Matrix Science Inc., Boston, MA, USA) for the MS/MS ion search.

The peptides from bands on SDS-PAGE gels were analyzed with Liquid Chromatography-Mass spectrometry using a Q Exactive mass spectrometer (Thermo Fisher Scientific Inc.) coupled to the UltiMate3000 RSL Cnano LC system (Dionex Co., Amsterdam, the Netherlands) with a nano HPLC capillary column, 150 mm × 75 μm i.d. (Nikkyo Technos Co., Tokyo, Japan), and a nanoelectrospray ion source. Reversed-phase chromatography was performed with a linear gradient (0 min, 5% B; 100 min, 40% B) of solvent A (2% acetonitrile with 0.1% formic acid) and solvent B (95% acetonitrile with 0.1% formic acid) at an estimated flow rate of 300 nl/min. A precursor ion scan was carried out using a 400–1600 mass to charge ratio (*m/z*) prior to MS2 analysis. MS2 scans were obtained for the 20 most intense peaks of each MS1 scan.

### Data analysis

The raw data were processed using Proteome Discoverer 1.4 (Thermo Fisher Scientific Inc.) in conjunction with the MASCOT search engine, version 2.6.0 (Matrix Science Inc., Boston, MA, USA) for protein identification. Peptides and proteins were identified using the human protein database in UniProt (release 2018_11), with a precursor mass tolerance of 10 ppm and a fragment ion mass tolerance of 0.02 Da. Fixed modification was set to carbamidomethylation of cysteine. Variable modifications were set to oxidation of methionine. Two missed cleavages by trypsin were allowed.

### Identification of POTEF sites recognized by TCP-1 complex proteins

Two truncated POTEF fragments were independently constructed into a pCMV6-Entry vector (OriGene). 293 T cells were seeded in 10-cm plates one day before and then each constructed plasmid DNA was transfected using Avalanche-Omni Transfection Reagent. One overnight after transfection, these cells were lysed. Western blotting and IP were then performed as described above.

### Immunofluorescence staining

CuO-POTEF-Flag HGrC1 cells were fixed with MeOH, permeabilized with 0.5% Triton X-100 in PBS, blocked in 1% bovine serum albumin (BSA)/PBS, and stained with the following antibodies: mouse: anti-TCP-1α (sc-374088), rabbit: anti-LC3 (PM036, MBL), and anti–DDDDK-tag mAb-Alexa Fluor 488 (M185-A48). Alexa Fluor-conjugated species-specific anti-IgG (Thermo Fisher Scientific) in 1% BSA/PBS was used as the secondary antibody. Images were obtained using BZ9000 microscopy (Carl Zeiss Microscopy Co., Ltd.). Secondary antibodies were conjugated with Alexa Fluor 594 for TCP-1α and for LC3. We set the colors of Alexa Fluor 488 to green and Alexa Fluor 594 to red. Nuclei were stained blue using ProLongGlass Antifade Mountant with NucBlue Stain (Invitrogen).

## Supplementary information

Supplemental figure 1

Supplemental figure 2

Supplemental figure 3

Supplemental table 1

Supplemental table 2

Supplemental table 3
