# Supplementary figures and images for: Primate-specific POTE-actin gene could play a role in human folliculogenesis by controlling the proliferation of granulosa cells
Source: Cell Death Discov. 2021 Jul 20;7:186. doi: 10.1038/s41420-021-00566-1 (PMC8292509; doi:10.1038/s41420-021-00566-1)

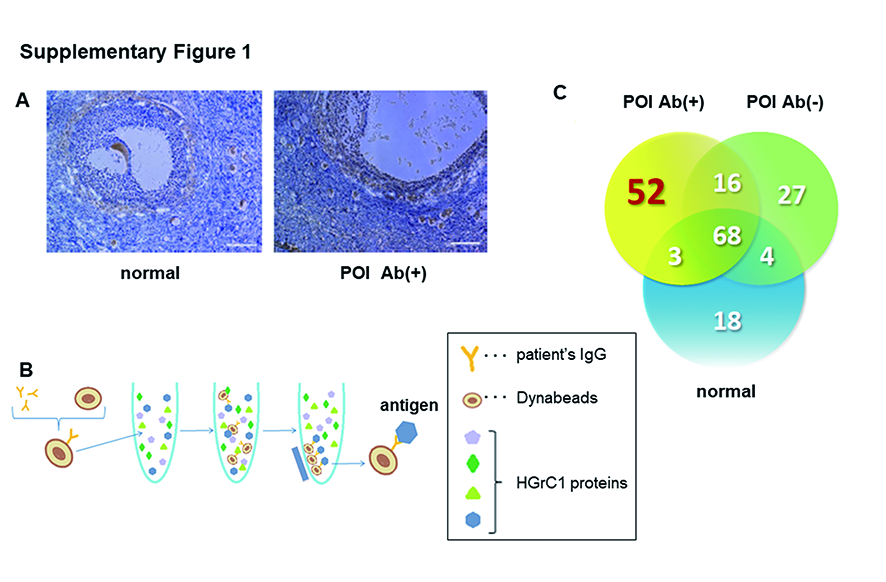

Supplement: Supplementary file 1 — Supplemental figure 1 [file 41420_2021_566_MOESM1_ESM.tif]

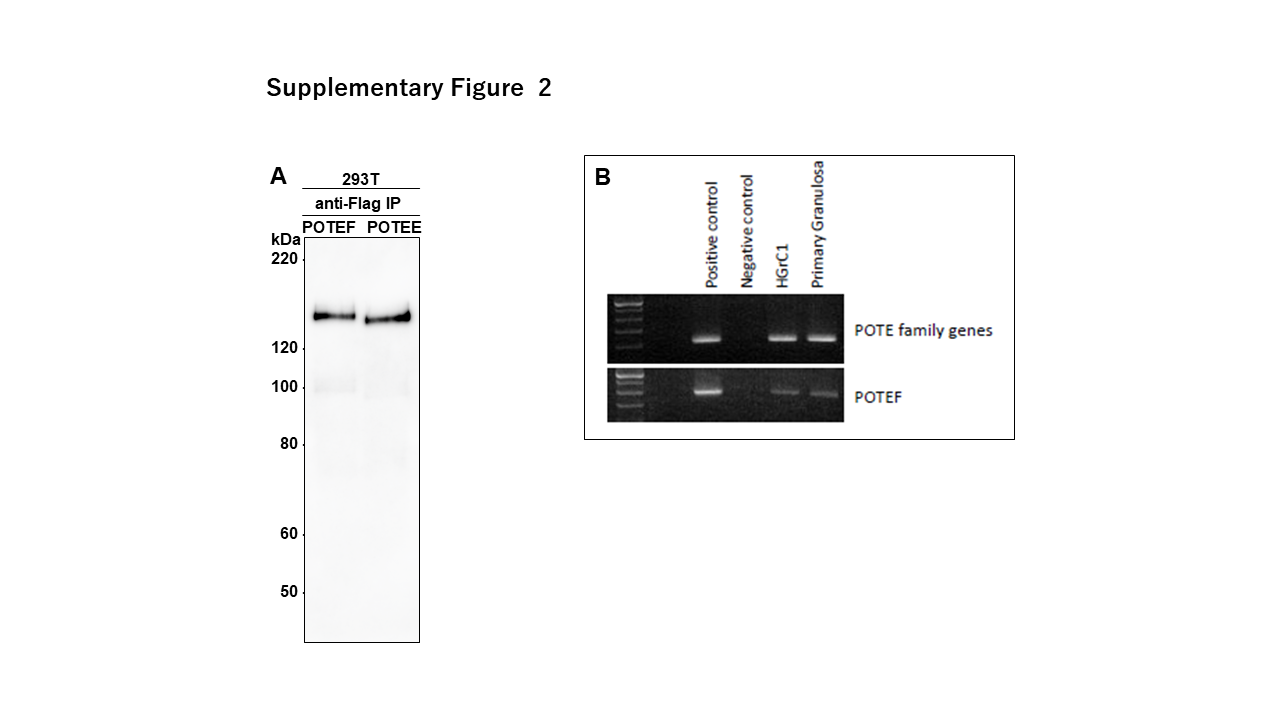

Supplement: Supplementary file 2 — Supplemental figure 2 [file 41420_2021_566_MOESM2_ESM.tif]

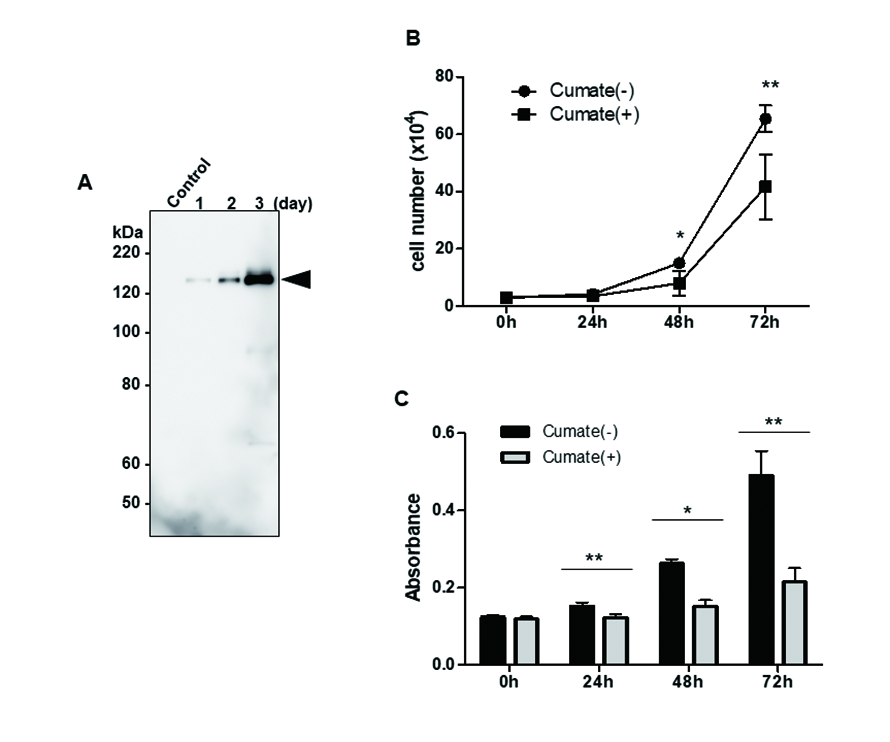

Supplement: Supplementary file 3 — Supplemental figure 3 [file 41420_2021_566_MOESM3_ESM.tif]
